# Predictors of participation in a telephone-based Acceptance and Commitment Therapy for smoking cessation study

**DOI:** 10.1186/s12889-015-2650-0

**Published:** 2015-12-23

**Authors:** Yim Wah Mak, Paul H. Lee, Alice Yuen Loke

**Affiliations:** School of Nursing, The Hong Kong Polytechnic University, Hung Hom, Hong Kong

**Keywords:** Smoking cessation program, Participation, Acceptance and commitment therapy

## Abstract

**Background:**

Little is known about factors that influence participation in smoking cessation trials among Chinese populations. The aim of this study is to explore the characteristics of individuals who chose to participate and those who chose not to participate in a proactive telephone-based acceptance and commitment therapy program for smoking cessation within a Chinese sample, and to identify predictors of program participation. Understanding the factors that predict participation in smoking cessation trials may allow researchers and healthcare professionals to target their recruitment efforts to increase the enrollment of smokers in smoking cessation programs.

**Methods:**

Participants were proactively recruited from six primary healthcare centers. Current cigarette smokers were screened for eligibility and then invited to complete a baseline questionnaire for the trial. The differences in characteristics between participants and non-participants as well as factors predictive of participation were analyzed using Chi-square tests and logistics regression.

**Results:**

A total of 30,784 clinic attendees were approached. From these, 3,890 (12.6 %) smokers were screened and identified. Of the 3,890 smokers, 420 (10.8 %) were eligible to participate and completed the baseline questionnaires. The analysis showed that participants (*n* = 142) and non-participants (*n* = 278) differed significantly in terms of demographics, smoking-related, and psychological variables. The following characteristics were found to predict program participation: those with a relatively high level of dependence on nicotine (OR = 3.75; 95 % CI = 1.25–11.23), those in the contemplation (OR = 7.86; 95 % CI = 2.90–21.30) or preparation (OR = 24.81; 95 % CI = 8.93–68.96) stages of change, and those who had abstained for one month or less in a previous attempt at quitting (OR = 3.77; 95 % CI = 1.68–8.47).

**Conclusions:**

The study shed light on the factors predictive of participation in a counseling-based smoking cessation program among a Chinese population. The results were encouraging, as most significant predictors (e.g., nicotine dependence, stage of change in smoking cessation) can be feasibly addressed or modified with interventions. No significant predictive relationships were found between psycho-social variables or socio-demographic variables and participation. Efforts should be made to increase the enrollment of smokers who are seemingly not yet ready to quit, and to tailor the program to fit the program’s participants.

## Background

Smoking has caused an enormous and avoidable public health catastrophe. Evidence has linked smoking to diseases of nearly every organ of the body, as well as to increases in healthcare utilization and costs [1, 2]. Smoking cessation has been found to lower the risk of developing smoking-related conditions, thus reducing healthcare costs [1]. Therefore, interventions to encourage people to stop smoking are essential. There are a number of pharmacological interventions [3] and behavioral approaches to support quitting [4, 5]. However, participation in smoking cessation treatments has been found to be modest, with less than one-third of smokers using counseling services and/or pharmacological treatments [6, 7]. Recruiting smokers to participate in smoking cessation studies is challenging, particularly those involving intensive, face-to-face treatments. Indeed, previous studies evaluating this type of approach found recruitment rates of less than 2 % of the targeted population [8, 9]. In addition, in these smoking cessation studies a reactive approach to recruitment was often used, with individuals being informed about the availability of an intervention program and having to initiate contact to participate. This approach only attracted smokers who were seeking treatment or motivated to quit, thus compromising the generalizability of the findings, as most smokers in the real world do not fall into this category [10]. A recent study showed that 74 % of their sample accepted a proactive offer to participate in telephone-based smoking cessation counseling, whereas only 9 % contacted the quit line themselves (reactive) and received telephone counseling [11]. Another study found that 80 % of smokers who were proactively “cold-called” by telephone participated in a smoking cessation intervention, suggesting that a proactive strategy is an effective method of recruiting smokers to participate in trials [12].

Although participation in a program may depend on the characteristics of the study, such as the type, format, and duration of treatment, as well as on recruitment strategies as suggested above, in a growing number of studies attention has also turned to the intrapersonal characteristics of participants and non-participants in randomized clinical trials (RCTs) of smoking cessation programs and to variables that influenced their participation [13–19]. The goal was to develop strategies to recruit smokers to participate in the program and to tailor the programs to maximize the effectiveness of the treatment. In general, in these studies it was found that compared with non-participants in smoking cessation programs, the participants had made more prior attempts to quit [17, 18], had a stronger intention or motivation to quit [13, 15, 16], and higher self-efficacy or greater confidence in their ability to quit [15, 17]. Nevertheless, findings of the differences between participants and non-participants have varied between studies, probably due to different measurement issues, types of interventions, recruitment approaches, and heterogeneous samples and settings. Only one of the studies used a proactive approach to explore the characteristics of the participants and non-participants as well as the predictors of participation in a telephone-based smoking cessation program. This is important, as most studies utilized a reactive approach to recruitment, and the study samples recruited through this approach might differ from proactively recruited samples. Also, so far, there is only one study that investigated issues of program participation in Asia, particularly among a Chinese sample [19]. We are therefore interested in exploring factors related to the participation of Chinese smokers in this brief, telephone-based program under proactive recruitment.

A third of the world’s smokers reside in mainland China, where it was reported in 2010 that 28.1 % of the population smoked daily [20]. Hong Kong, however, has a different set of tobacco control policies, resulting in an overall daily cigarette smoking prevalence rate of 10.7 % in 2012 [21]. For instance, in Hong Kong smoking is prohibited in designated non-smoking areas in various indoor public spaces, such as indoor workplaces and schools [22]. In addition, several smoking cessation programs targeting different groups of smokers are currently being offered [23]. It is therefore important to run this proactive telephone-based ACT program among this Chinese population to target smokers that the reactive programs do not reach.

In addition, information about non-participants is crucial for interpreting the results of cessation trials and evaluating the generalizability and external validity of the findings, as well as the feasibility of this telephone-based smoking cessation program [24].

The aim of this study is to describe and compare the characteristics of both individuals who chose to participate and those who chose not to participate in a proactive telephone-based Acceptance and Commitment Therapy (ACT) program on smoking cessation among a Chinese population, and to identify the predictors of program participation. We examine variables that were shown to be important in previous smoking cessation studies, including the intention to quit, the level of dependence on smoking, self-efficacy, social support, smoking history variables, current smoking status, whether there is other smoker in the household, and the socio-demographic characteristics of the participants. We included socio-demographic characteristics such as gender, age, education level, marital status, employment status, household income and whether the participant had a current smoking partner in order to explore whether these factors affected participation in this smoking cessation program. Previous studies [25, 26] found that the male gender, older age, higher income, and the absence of other smokers in the household were predictors of participation in a smoking cessation program; whereas nicotine dependence was more strongly associated with cessation than with measures of motivation [25, 26]. We also included a broader range of variables such as perceived health status and the use of emotion regulation strategies in order to explore whether these factors also affected participation in this smoking cessation program. Previous studies [27, 28] found that the majority of smokers reported health issues as the main reason for quitting. It was therefore hypothesized that those who perceived themselves to have poorer health would be more likely to join the smoking cessation program. With regard to strategies used to regulate emotions, these can be broadly categorized into reappraisal techniques and suppression strategies [29]. Expressive suppression, that is, inhibiting the expression of ongoing emotions once they have been generated, has been found to be associated with greater negative affect and more cravings [28–31]. It was therefore predicted that those with higher expressive suppression scores would be more likely to join the program to seek professional help to quit smoking, as such people are more capable of resisting the urge to smoke. On the other hand, cognitive reappraisal, that is, reevaluating a situation to influence its emotional impact, was associated with less cravings and lower expectations that smoking would reduce the negative affect [30, 32]. Therefore, it was predicted that individuals with higher cognitive reappraisal scores would be less likely to participate in the program than those with lower scores, as quitting smoking might be relatively easier for the former.

## Methods

### Participants and procedures

This study was based on baseline data collected as part of a randomized control trial (RCT) examining the potential efficacy and feasibility of ACT for smoking cessation [33]. Participants were proactively recruited from July 2012 to December 2013 from six primary healthcare centers where general practitioners were available for consultations or healthcare screenings. All of the centers were community health centers providing health checks and health maintenance and treatment services. Attendees of the centers were approached and smokers were identified through eligibility screening with the help of the clinic’s staff, and then cross-checked by research assistants. Inclusion criteria included the following: (1) aged 18 years or above; (2) smoked at least one cigarette per day in the past 30 days; (3) not currently participating in any other smoking cessation program; (4) able to communicate in Cantonese; (5) Hong Kong residents; (6) currently residing in Hong Kong and expecting to continue to do so for the next 6 months; and (7) have access to a telephone. Individuals who were eligible were invited to complete a baseline questionnaire. The self-administered questionnaires were distributed and collected in the healthcare centers while the individuals waited to receive services. Eligible participants who gave their written consent to participate in the study were randomized to either the ACT or the control group. The randomization process was based on computer-generated randomization numbers placed in sealed and opaque envelopes. Each envelope had a serial number written on it. Each was then opened by the research assistant in accordance with the sequence in which the participants were admitted at the respective centers. The randomization procedure was undertaken by another research assistant who was not directly involved in the study.

### Measures

The baseline questionnaire solicited information on the participants’ self-perceived health status, assessed using the Short Form-12 Health Survey (SF-12) [34]; current smoking status and behavior; level of nicotine dependence as measured by the Fagerstrom Test for Nicotine Dependence [35]; quitting history; intention to quit based on Prochaska’s model [36]; and level of cognitive reappraisal and expressive suppression as measured by the Emotion Regulation Questionnaire (ERQ) [29]. Their perception of the importance and difficulty of quitting, as well as their confidence in being able to quit, were measured using a 10-point scale ranging from 0 (not important/difficult/confident at all) to 100 (very important/difficult/confident). The demographic information of the participants was also collected, including details of their age, gender, marital status, educational attainment, occupation, monthly household income, and the number of people in their household. A detailed description of the questionnaire can be found elsewhere [33].

With regard to smoking status, the participants were categorized as daily smokers (smoke 1 or more cigarettes per day or 7 or more cigarettes per week), occasional smokers (smoke less than 7 cigarettes per week), and those who recently quit smoking (stopped for 7 days but not more than 1 month preceding the survey) [37]. Ex-smokers who had not smoked for more than 1 year were treated as former-smokers, and were thus not eligible to participate in this study.

Details on the type of information provided during the intervention can be found elsewhere [33].

### Ethical considerations

Ethical approval for the study was obtained from the Human Subjects Ethics Application Review Committee of The Hong Kong Polytechnic University. Written consent was obtained from the participants. Participation was voluntary under the assurance of confidentiality.

### Statistical analyses

Statistical analyses were carried out using the Statistical Package for Social Sciences (SPSS) version 21.0 [38]. All tests were two tailed with a significance level of *p* < .05. Independent sample t tests were used to examine mean differences in continuous variables, and Chi-square tests were used to examine differences in proportions for categorical variables. Summary statistics included means and standard deviations for continuous variables and frequencies and percentages for categorical variables. Significant variables in the univariate analysis were simultaneously entered into the logistic regression model by using a “ENTER” procedure to identify the factors predictive of participation. The goodness-of-fit for the model was assessed by the Hosmer-Lermershow test, where *p* > 0.05 indicates an acceptable fit. The estimated adjusted odds ratios and 95 % confidence intervals (CI) of all of the predictive factors in the logistic regression model were reported. Expectation-Maximization (EM) followed by a multiple imputation procedure was applied to impute the missing data [39].

## Results

A total of 30,784 subjects were approached and 3,890 (12.6 %) were identified as smokers through preliminary screening. Of the 3,890 smokers, 420 (10.8 %) completed the baseline questionnaires. The baseline assessment data consisted of 142 (33.8 %) participants and 278 non-participants.

The three main reasons reported by the non-participants for not taking part in the study were: want to rely on oneself or believe they can quit smoking on their own (*n* = 25, 9.0 %); had no time or time does not fit with the study schedule (*n* = 20, 7.2 %); and not interested in quitting smoking or in the project (*n* = 15, 5.4 %). (See Table 1.) Compared with the general Hong Kong population [40], we recruited more males and middle-aged and older adults, and fewer of them had been educated to the level of matriculation or above (Table 2).

**Table 1:** Reasons for not taking part in the study

Reasons	Non-participants
	(*n* = 278)
Want to rely on self/can quit on one’s own	25 (9.0 %)
No time/time does not fit with study schedule	20 (7.2 %)
Not interested in quitting	15 (5.4 %)
Do not believe in counseling	5 (1.8 %)
Lack of determination to quit	4 (1.4 %)
Fear of uncomfortable feelings when quitting	3 (1.1 %)
Troublesome	2 (0.7 %)
Need to smoke for social contact	2 (0.7 %)
No need to quit now	2 (0.7 %)
Don’t want to talk to a researcher	1 (0.4 %)
Need to rely on smoking to concentrate	1 (0.4 %)
Old age	1 (0.4 %)
Previous attempt to quit ended in failure	1 (0.4 %)
Did not provide reasons	197 (70.9 %)

**Table 2:** Differences in socio-demographics between the participants and non-participants at baseline (*n* = 420)

	Hong Kong general population in 2011	Non-participants (*N* = 278)		Participants (*N* = 142)			
Variable	%	n	%	n	%	*X* ^2^ *(df)*	*p*
Gender						8.26 (1)	.004**
Male	46.7	228	83.2	101	71.1		
Female	53.3	46	16.8	41	28.9		
Age						11.80 (2)	.003**
18–35 (Young adults)	25.7	46	16.9	38	27.0		
36–55 (Middle-aged)	34.4	118	43.4	69	48.9		
56 or above (Older adults)	24.7	108	39.7	34	24.1		
Educational attainment						3.28 (2)	.194
Primary or below	29.3	71	26.3	31	22.0		
Secondary or below	41.4	158	58.5	95	67.4		
Matriculation or above	29.3	41	15.2	15	10.6		
Marital Status						1.51 (2)	.471
Single	39.6	42	15.5	27	19.6		
Married	51.0	203	74.9	101	73.2		
Divorced/separated/widowed	9.4	26	9.6	10	7.2		
Employment status^a^						7.95 (1)	.005**
Currently employed	58.1	184	69.2	115	82.1		
Unemployed	41.9	82	30.8	25	17.9		
Monthly household income^b^						3.20 (2)	.202
HK$9,999 or less	23.8	44	18.3	22	16.1		
HK$10,000-29,999	41.4	114	47.5	78	56.9		
HK$30,000 or above	34.8	82	34.2	37	27.0		
Living with others						.01 (1)	.910
Yes	82.9	244	90.4	127	90.7		
No	17.1	26	9.6	13	9.3		
With a current smoking partner						9.38 (1)	.002**
Yes	-	32	11.7	33	23.2		
No	-	241	88.3	109	76.8		

Note: The sample sizes per variable may not add up to 420 because of missing values

*p < =.05, **p < =.01, ***p < =.001 by *X* ^2^ test

^a^Includes individuals who are currently employed, housewives, and full-time students

^b^US$1 = HK$7.8

df = degree of freedom

### Differences in the characteristics of the participants and non-participants

Table 2 summarizes the socio-demographic characteristics of the participants and non-participants in the smoking cessation study who completed the baseline questionnaire. A significantly greater number of participants than non-participants were female (28.9 % vs. 16.8 %, *p* = .004), younger adults (27.0 % vs. 16.9 %), employed (82.1 % vs. 69.2 %, *p* = .005) and had a partner who smoked at the time of the assessment (23.2 % vs. 11.7 %, *p* = .002). The two groups did not differ in marital status, level of education, living status, and monthly household income.

With regard to smoking behavior and history (Table 3), the participants and non-participants did not differ in years of smoking, smoking habit at home (yes vs no), social support in relation to quitting, and whether or not there had been any previous attempt to quit. In addition, significantly more participants than non-participants had abstained for one month or less in a previous attempt at quitting (68.2 % vs. 43.0 %, *p* = <.001); had a high level of nicotine dependence (36.6 % vs. 15.3 %); and were daily smokers (i.e., smoked 1 or more cigarettes per day or 7 or more cigarettes per week) (99.3 % vs. 93.9 %). Participants consumed significantly more cigarettes per day (i.e., smoked 21 or more cigarettes) in the past month compared to non-participants (16.2 % vs. 9.4 %). There was also a significant difference between participants and non-participants in their intention to quit according to Prochaska’s stages of behavioral change (*p* < .001). Participants were significantly more likely than non-participants to be in the contemplation (29.6 % vs. 12.6 %) and preparation stages of quitting smoking (51.1 % vs. 9.3 %), whereas non-participants were more likely to be in the pre-contemplation stage (74.8 % vs. 15.6 %).

**Table 3:** Differences in smoking characteristics and intention to quit between participants and non-participants at baseline (*n* = 420)

Current smoking status and behavior	Non-participants (*N* = 278)		Participants (*N* = 142)			
Variable	n	%	n	%	*X* ^2^ *(df)*	*p*
Smoking status^a^					7.10 (2)	.029*
Daily smoker	261	93.9	141	99.3		
Occasional smoker	11	4.0	0	0		
Recently quit smoking	6	2.2	1	0.7		
Average cigarettes/day in the past 1 month					8.09 (2)	.018*
10 or less	133	48.2	50	35.2		
11–20	117	42.4	69	48.6		
21 or more	26	9.4	23	16.2		
Years of smoking					4.94 (3)	.176
1–10	19	7.1	11	7.8		
11–20	53	19.9	39	27.7		
21–30	50	18.8	30	21.3		
31 or more	144	54.1	61	43.3		
Nicotine dependence level^b^					24.29 (2)	<.001***
Low	154	56.2	61	43.0		
Moderate	78	28.5	29	20.4		
High	42	15.3	52	36.6		
Smoke at home					3.80 (1)	.051
Yes	201	73.1	116	81.7		
No	74	26.9	26	18.3		
Social support in quitting					.59 (1)	.443
Yes	259	93.2	135	95.1		
No	19	6.8	7	4.9		
Quitting history						
Previous attempt at quitting					2.34 (1)	.126
No	121	43.7	51	35.9		
Yes	156	56.3	91	64.1		
Length of abstinence in last attempt at quitting^c^					14.08 (1)	<.001***
A month or less	65	43.0	60	68.2		
More than a month	86	57.0	28	31.8		
Stages of quitting smoking					139.65 (3)	<.001***
Pre-contemplation	202	74.8	21	15.6		
Contemplation	34	12.6	40	29.6		
Preparation	25	9.3	69	51.1		
Action	9	3.3	5	3.7		

Note: The sample sizes per variable may not add up to 420 because of missing values

*p < =.05, **p < =.01, ***p < =.001 by *X* ^2^ test

^a^Smoking status was categorized as daily smoker (smokes 1 or more cigarettes per day or 7 or more cigarettes per week), occasional smoker (smokes less than 7 cigarettes per week), and recently quit smoking (stopped for 7 days but not more than 1 month preceding the survey)

^b^Measured by the Fagerstrom scale, which is divided into 3 levels: low (0–3), moderate (4–5), and high (6–10)

^c^Only individuals who had made past attempts to quit were required to answer this question

With regard to self-perception in quitting (Table 4), participants perceived greater importance in quitting (M = 70.5, SD = 23.0 vs. M = 57.1, SD = 27.7, *p* < .001) and greater difficulty in quitting (M = 67.8, SD = 25.7 vs. M = 57.9, SD = 29.1, *p* < .001) than non-participants. Participants and non-participants were similar in their perceived physical and mental health status, confidence in being able to quit, as well as in their level of cognitive reappraisal and expressive suppression.

**Table 4:** Differences in psychological variables and perceived health status between participants and non-participants at baseline (*n* = 420)

	Non-participants (*N* = 278)		Participants (*N* = 142)			
	Mean	SD	Mean	SD	*T, df*	*p*
Self-perception of quitting						
Importance of quitting^a^	57.1	27.7	70.5	23.0	t = −4.91 df = 405	<.001***
Difficulty in quitting^b^	57.9	29.1	67.8	25.7	t = −3.39 df = 403	<.001***
Confidence in being able to quit^c^	54.0	30.1	55.7	23.8	t = −0.61 df = 344.6	.541
Perceived health status^d^						
Physical Component Summary (PCS)	45.8	9.9	46.1	8.0	t = −0.21 df = 346	.837
Mental Component Summary (MCS)	49.4	11.9	47.9	9.9	t = 1.13 df = 346	.260
ERQ						
Cognitive reappraisal	8.8	1.7	8.6	1.6	t = .1.11 df = 317	.267
Expressive suppression	9.5	2.1	9.7	1.9	t = −0.70 df = 323	.485

*p < =.05, **p < =.01, ***p < =.001 by independent sample t-tests

^a^Based on a 10-point scale ranging from 0 (not important at all) to 100 (very important)

^b^Based on a 10-point scale ranging from 0 (not difficult at all) to 100 (very difficult)

^c^Based on a 10-point scale ranging from 0 (no confidence at all) to 100 (very confident)

^d^Measured by the Short Form-12 Health Survey (SF-12). The PCS and MCS scores have a range of 0 to 100 and were designed to have a mean score of 50 and a standard deviation of 10 in a representative sample of the US population

### Predictors of participation

The results of the logistic regression model are shown in Table 5. Three variables significantly predicted participation in the smoking cessation study. Those who had abstained for one month or less in a previous attempt at quitting were 3.77 times as likely to join the program (OR = 3.77, CI = 1.68–8.47). Smokers who were in the preparation (OR = 24.81, CI = 8.93–68.96) and contemplation stages (OR = 7.86, CI = 2.90–21.30) were near 24 times and 7 times respectively more likely to join the program than those in the pre-contemplation stage. Having a high level of nicotine dependence was associated with more than three-fold increase in program participation (OR = 3.75, CI = 1.25–11.23). No other significant predictor of program participation was found.

**Table 5:** Summary of the logistic regression model in predicting participation (“ENTER” method is used)

Independent variables	Crude OR	(95 % CI)	Adjusted OR^a^	(95 % CI)
Gender (ref = male)				
Female	2.01	1.24–3.26**	1.00	0.34–2.93
Age (ref = 56 or above)				
18–35	2.62	1.47–4.67**	2.02	0.59–6.92
36–55	1.86	1.14–3.02*	1.36	0.50–3.71
Employment status (ref = unemployed)				
Currently employed	2.05	1.24–3.40**	0.99	0.32–3.04
With a current smoking partner (ref = no)				
Yes	2.28	1.33–3.90**	2.71	0.80–9.15
Average cigarettes/day in past 1 month (ref = 1–20)				
21 or above	1.86	1.02–3.39*	1.36	0.38–4.92
Nicotine dependence level (ref = low)^b^				
Medium	0.94	0.56–1.58	1.53	0.60–3.89
High	3.13	1.89–5.17***	3.75	1.25–11.23*
Length of abstinence in last attempt at quitting (ref = More than a month)				
A month or less	2.84	1.63–4.93***	3.77	1.68–8.47**
Stages of change (ref = Pre-contemplation)				
Contemplation	11.32	5.96–21.48***	7.86	2.90–21.30***
Preparation	26.55	13.98–50.42***	24.81	8.93–68.96***
Action	5.34	1.64–17.43**	2.85	0.62–13.18
Importance of quitting^c^ (mean = 60) (ref = more important)				
Less important	0.40	0.26–0.61***	0.53	0.23–1.22
Difficulty in quitting^d^ (mean = 60) (ref = more difficult)				
Less difficult	0.66	0.44–1.00*	1.31	0.59–2.89

*p < =.05, **p < =.01, ***p < =.001

*OR* odds ratio; *CI* confidence interval

Note: The numbers in italics are the significant results and their 95 % confidence intervals

^a^Adjusted for all of the significant variables in the univariate analysis

^b^Measured by the Fagerstrom scale, which is divided into 3 levels: low (0–3), moderate (4–5), and high (6–10)

^c,d^These variables were classified as low versus high by placing the division at the mean value

## Discussion and conclusions

This study explored a broad range of differences in individual characteristics between those who chose to participate and those who chose not to participate in the proactive telephone-based ACT program for smoking cessation, and examined the predictors of program participation. Several measures were found to differentiate the participants from the non-participants, including age, gender, employment status, whether or not their partner was a smoker at the time of the assessment, current smoking status and cigarette consumption, level of nicotine dependence, length of abstinence in a previous attempt at quitting, intention to quit, the perceived importance and difficulty of quitting, daily consumption of cigarettes, and smoking habit. Smokers who had a higher level of nicotine dependence, were in the preparation stage of change, and had a current smoking partner at the time of the assessment were found to be likely to participate in the program.

Our findings were mostly comparable to those of previous studies on program participation [13–19]. In terms of smoking habits or behaviors, smokers who smoked at home and with a current smoking partner were more likely to participate in the program. It was speculated that smoking at home and having more smokers in the family might cause smokers greater concern about the impact of smoking on the health of their family. The results also showed that those who joined the program tended to be daily smokers and had a higher level of nicotine dependence than those who declined. Higher nicotine dependency also predicted program participation. Nicotine dependency was believed to be a barrier to quitting smoking, and those who were more dependent might have perceived quitting smoking to be more difficult. Hence, they might have wanted to get help from professionals in the process of quitting. As daily smoking was found to be related to nicotine dependence [41], it made sense that the more nicotine-dependent participants would also have been more eager than the other participants to receive professional help in quitting.

With respect to their intention to quit, our finding was consistent with previous studies [13, 15] indicating that smoking cessation intervention studies often only reach those smokers who are “ready” to participate in such programs or who are at the “contemplation” or “action” stages of quitting. The implications of this are that rather than waiting for individuals to seek smoking cessation treatment, it may be important to set up programs directed at individuals in the earliest stages of quitting smoking, to help them move through the stages of change or to modify their motivation and attitude towards smoking, so as to encourage them to be interested in participating in smoking cessation programs.

In terms of self-perception in quitting, participants in the program perceived quitting as being more important than non-participants did, which made sense, as this is what caused the participants to join the program. Previous studies found that participants had more confidence in their ability to quit than non-participants did [15, 17], but this was not observed in our study. It is possible that having the confidence to quit was more relevant to participation in programs that were relatively costly to join. As our study was free and required little commitment in terms of time, individuals might have regarded the program was “worth trying,” regardless of their level of confidence in quitting.

The results showed that there was no difference between participants and non-participants in the use of expressive suppression and cognitive reappraisal strategies when regulating their emotions. One possible reason for this was that the ERQ measured the ability of individuals to regulate their emotions in life in general but not specifically their emotions in relation to smoking. More research should be conducted in the future to investigate whether reappraisal and suppression scores are predictive of the actual use of these strategies by smokers.

With regard to program recruitment, less than 40 % of smokers who were screened agreed to take part in the study, which was lower than the participation rate reported in previous studies in which a proactive approach was also used to recruit smokers to take part in smoking cessation trials (67 to 80 %) [11, 12, 37]. Yet the uptake rate for this study was still a few times higher than for reactive telephone counseling (9 %) [11], as well as for counseling (2.9 %) and nicotine replacement therapy (11.7 %), as found in a population-based study [6]. This shows that a proactive strategy is a useful way of increasing the proportion of smokers recruited into treatment. The lower participation rate in this study compared to studies that also used a proactive approach to recruitment might be because most of our smokers were proactively recruited from health clinics. These individuals might attend health clinics for check-ups rather than seek medical consultations for immediate health issues. In this sense, this group of smokers might be relatively healthy and might not have thought of quitting smoking.

However, one point to note was that the perceived health of both groups was slightly below average, which did not support our hypothesis that those who joined the program perceived their health to be worse than those who did not join. It is possible that although these smokers might not have been diagnosed with smoking-related diseases or have significant health problems, there might already be some signs and symptoms of deterioration in their functioning, as reflected in their self-reported perceived health status, of which the individuals might not have been aware. To motivate them to quit smoking, drawing the attention of individuals to their perceived health status could be a useful first step, followed by increasing their awareness of the perceived susceptibility of their health to threats from smoking and of how quitting can reverse some of that damage to their health. Without recognizing the risks involved in smoking, it is unlikely that these people would attempt to quit smoking. Furthermore, although the use of proactive recruitment enabled us to reach smokers who would not otherwise have initiated smoking cessation on their own, less than 4 % of the smokers who were approached eventually agreed to participate in this study. This has implications for the feasibility of the study and for future clinical studies and practice.

We found that smokers who had a higher level of dependence on nicotine, were in the preparation stage of change, and had a current smoking partner at the time of the assessment were more likely to participate in the program. In the present study, with assistants from general practitioners and nurses in the clinics to identify smokers and refer them to the program, it was feasible for us to recruit smokers proactively in primary health settings. Therefore, for a more cost-effective way of recruiting participants in the future, we suggest that general practitioners, nurses, or clinical staff be encouraged to routinely identify and refer all people who smoke to a smoking cessation program. In addition, smoking cessation programs could focus on lowering the level of nicotine dependence and moving participants from the pre- contemplation and contemplation stages to the preparation stage of quitting smoking.

The current study has several limitations. First, although every effort was made to encourage smokers to complete the baseline questionnaire, only around 10 % of the smokers who were approached did so. No data were available on participants who refused to complete the questionnaire, their reasons for not completing the questionnaire, and their reasons for not participating in the study. This could limit the generalizability of the findings and potentially lead to sample bias. The low participation rate in smoking cessation programs by individuals in Hong Kong and other Chinese cities may hinder existing programs. A fuller set of qualitative data on reasons for non-participation, barriers to participation, as well as suggestions for making participation in smoking cessation programs more likely may provide us with richer information on program accessibility and inform us of ways to promote smoking cessation in this population. Second, although a broad range of variables were included in the study for a more comprehensive approach to participation, the features of the program as well as factors that might have influenced participation (e.g., mistrust of a research-based program, skepticism of the efficacy of counseling), were not assessed. Future research in these areas will be worthwhile.

Despite the above limitations, this study enriches the body of literature on the characteristics of participants and non-participants in a proactive telephone-based program on smoking cessation using a proactive approach towards a Chinese population. The study also shed light on the factors predictive of participation in a smoking cessation program among a Chinese population. The results were encouraging, as most significant predictors (e.g., nicotine dependence, intention to quit) can be feasibly addressed or modified with interventions. The differences that were observed will also enable healthcare professionals to understand who comprises the target population so as to achieve two main purposes: (1) to tailor programs to those who are likely to join programs, so as to maximize the effectiveness of the programs; and (2) most importantly, to refine recruitment and intervention efforts to target those who are less likely to take part in smoking cessation programs in order to increase enrollment of underserved smokers and, ultimately, to lower the prevalence of smoking.
